# Conditioned Medium From Azurin-Expressing Human Mesenchymal Stromal Cells Demonstrates Antitumor Activity Against Breast and Lung Cancer Cell Lines

**DOI:** 10.3389/fcell.2020.00471

**Published:** 2020-07-09

**Authors:** Marília Silva, Gabriel Amaro Monteiro, Arsenio M. Fialho, Nuno Bernardes, Cláudia Lobato da Silva

**Affiliations:** iBB-Institute for Bioengineering and Biosciences, Department of Bioengineering, Instituto Superior Técnico, Universidade de Lisboa, Lisbon, Portugal

**Keywords:** mesenchymal stromal cells, secretome, azurin, cancer, gene delivery

## Abstract

Recently, cell-based therapies have been explored as a strategy to enhance the specificity of anticancer therapeutic agents. In this perspective, human mesenchymal stromal cells (MSC) hold a promising future as cell delivery systems for anticancer proteins due to their unique biological features. In this study, we engineered human MSC to secrete a human codon-optimized version of azurin (hazu), a bacterial protein that has demonstrated anticancer activity toward different cancer models both *in vitro* and *in vivo*. To this end, microporation was used to deliver plasmid DNA encoding azurin into MSC derived from bone marrow (BM) and umbilical cord matrix (UCM), leading to expression and secretion of hazu to the conditioned medium (CM). Engineered hazu-MSC were shown to preserve tumor tropism toward breast (MCF-7) and lung (A549) cancer cell lines, comparable to non-modified MSC. Azurin was detected in the CM of transfected MSC and, upon treatment with hazu-MSC-CM, we observed a decrease in cancer cell proliferation, migration, and invasion, and an increase in cell death for both cancer cell lines. Moreover, expression of azurin caused no changes in MSC expression profile of cytokines relevant in the context of cancer progression, thus suggesting that the antitumoral effects induced by hazu-MSC secretome might be due to the presence of azurin independently. In conclusion, data shown herein indicate that MSC-produced azurin in a CM configuration elicits an anticancer effect.

## Introduction

Human mesenchymal stromal cells (MSC) are multipotent cells with the ability to modulate several biological mechanisms through paracrine activity (Hofer and Tuan, 2016), namely, limiting apoptosis (Gao et al., 2016) and inducing angiogenesis (Brewster et al., 2018; Mathew et al., 2019; Rifai et al., 2019), as well as to differentiate into a variety of cell lineages, including osteocytes, adipocytes, and chondrocytes (Xie et al., 2013). Cells with these features hold a promising future for cell therapies and tissue engineering, by potentially replacing damaged tissues of mesodermal origin and promoting tissue regeneration,. As such, the number of clinical trials using MSC has been rising almost exponentially since 2004 (Murray and Péault, 2015), achieving a total of 916 studies in 2020 (data from clinicaltrials.gov/, accessed on February 29th, 2020, using the terms “mesenchymal stem cell OR mesenchymal stromal cell”), of which 269 have been completed to date. In addition, MSC show an intrinsic ability to specifically migrate toward pro-inflammatory microenvironments, such as tumor sites (Kim et al., 2016; Cao et al., 2018; Chulpanova et al., 2018). This phenomenon occurs through an intricate crosstalk of biochemical cues, and although the underlying mechanisms are still not fully elucidated in this process, it has been recognized that the C-XC chemokine receptor type 4 (CXCR4)–stromal cell-derived factor 1 (SDF1α) axis plays an important role (Song and Li, 2011; Bhoopathi et al., 2012; Ho et al., 2014). Taking advantage of their innate tropism for tumors, genetically engineered versions of MSC have been under preclinical and clinical development as cell delivery systems of several anticancer agents. One of the most commonly adopted approach is the enhancement of endogenous antitumor immunity by engineering MSC to produce antitumor cytokines or soluble factors such as β-interferon (Ahn et al., 2013; Dembinski et al., 2013; Chen et al., 2019), interleukin-2 (Mounayar et al., 2013; Zhao, 2013), interleukin-12 (Elzaouk et al., 2006; Jeong et al., 2015), interleukin-15 (Jing et al., 2014), INF-alpha (Ren et al., 2008), or CX3CL1 (Xin et al., 2007). Another approach is the use of MSC to deliver tumor cytotoxic agents such as TRAIL (TNF-α related apoptosis inducing ligand) (Grisendi et al., 2010; Loebinger et al., 2010; Deng et al., 2014; Yan et al., 2014; Xia et al., 2015; Rossignoli et al., 2019; Spano et al., 2019), osteoprotegerin (OPG) (Qiao et al., 2015), NK4 (Kanehira et al., 2007), and HGF (Zhu et al., 2009). The employment of MSC as gene-directed enzyme-producing vehicles, such as MSC expressing thymidine kinase of the Herpes simplex virus with ganciclovir as a prodrug (tkHSV-MSC/GCV system) (Matuskova et al., 2012) and MSC engineered to express fused yeast cytosine deaminase::uracil phosphoribosyl transferase (yCD::UPRT) with 5-fluorocytosine (5-FC) as a prodrug (yCD::UPRT-MSC/5FC system) (Ursula et al., 2019), has also demonstrated very promising results. Three first-in-human clinical trials assessing gastrointestinal cancer, lung cancer, and ovarian cancer are being conducted to investigate the efficacy of genetically modified MSC in cancer patients with results demonstrating safety and tolerability, and some preliminary signs of efficacy (von Einem et al., 2019).

Azurin, a small water-soluble (14-kDa) protein from the bacteria *Pseudomonas aeruginosa*, has been explored in what concerns its antitumoral capacity. Azurin is able to enter mammalian cells, preferentially cancer cells (Bizzarri et al., 2011; Bernardes et al., 2018), acting at the membrane level by increasing its permeability and attenuating proliferative signaling pathways (Bernardes et al., 2014, 2016). After internalization, azurin forms a complex with the tumor suppressor protein p53, stabilizing it, and increasing its concentration at the intracellular level, thereby inducing apoptosis (Yamada et al., 2004). Azurin is also described to be able to increase the effectiveness of conventional anticancer therapeutics such as doxorubicin and paclitaxel (Bernardes et al., 2018), and gefitinib or erlotinib (Bernardes et al., 2016). In addition, a peptide derived from this protein (named p28) also enhances the activity of DNA damaging chemotherapeutic agents (Yamada et al., 2016). Azurin and p28 have a complex mechanism of action targeting several independent signaling pathways relevant in tumor proliferation, while inducing reduced side effects *in vitro* and *in vivo* (Lulla et al., 2016). These features turn azurin/p28 distinct and promising relatively to other antitumor agents, which have a more limited range of action.

In the present study, we couple azurin’s antitumoral effect to the tumor tropism ability of MSC, in a cell-based approach, by genetically engineering human MSC to produce and secrete azurin through non-viral methods. Though viral systems have demonstrated the highest gene transfer efficiencies in preclinical and clinical trials, non-viral vectors and gene transfer approaches are emerging as safer and effective alternatives. In this context, we employ a non-viral method, previously developed by our group, of human MSC transfection through microporation aiming at a high gene delivery efficiency, without compromising cell viability and recovery (Madeira et al., 2011).

When evaluating the role of naïve MSC in tumor progression/suppression, the majority of studies employ MSC isolated from the BM, the UCM, and the adipose tissue (AT) (Rahmatizadeh et al., 2019; Liang et al., 2020; Xia et al., 2020). Considering that MSC isolated from different tissue sources express different surface markers (Hass et al., 2011; Elahi et al., 2016), and may differ in what concerns differentiation potential (Rebelatto et al., 2008), the outcome from these studies may be dependent on the isolation source of MSC. Therefore, in the present study, all experiments were validated with MSC from two tissue sources, BM and UCM. Moreover, envisaging the translational potential of our approach, this study was performed under xenogeneic (xeno)-free culture conditions to avoid the batch-to-batch variations associated with the use of animal-derived products, allowing a better reproducibility and preventing contagious health risks from animal-derived viral agents, mycoplasma, and prions (Leong et al., 2016).

## Materials and Methods

### Cell Lines and Cell Cultures

Cancer cell lines A549 (lung) and MCF-7 (breast) were obtained from ECACC (European Collection of Authenticated Cell Cultures) and cultured using high glucose Dulbecco’s modified Eagles’ medium (DMEM) supplemented with 10% of heat-inactivated fetal bovine serum (FBS) (Lonza), 100 IU/ml penicillin, 100 mg/ml streptomycin (PenStrep, Invitrogen), and passaged between 2 and 3 times per week, by chemical detachment with trypsin 0.05%.

Human MSC used in this study are part of the cell bank available at the Stem Cell Engineering Research Group (SCERG), Institute for Bioengineering and Biosciences at Instituto Superior Técnico (iBB-IST). MSC were previously isolated/expanded according to protocols previously established at iBB-IST (Santos et al., 2009; Soure et al., 2016). Originally, human tissue samples were obtained from local hospitals under collaboration agreements with iBB-IST (bone marrow: Instituto Portugu\^text{e}s de Oncologia Francisco Gentil, Lisbon; umbilical cord: Hospital São Francisco Xavier, Lisbon, Centro Hospitalar Lisboa Ocidental, Lisbon). All human samples were obtained from healthy donors after written informed consent according to the Directive 2004/23/EC of the European Parliament and of the Council of 31 March 2004 on setting standards of quality and safety for the donation, procurement, testing, processing, preservation, storage, and distribution of human tissues and cells (Portuguese Law 22/2007, June 29), with the approval of the Ethics Committee of the respective clinical institution. Human MSC from the different tissue sources (BM and UCM) were kept cryopreserved in a liquid/vapor-phase nitrogen container. Upon thawing, cells were cultured in StemPro^®^ Serum-free (SFM) medium and passaged two times per week, by chemical detachment with TrypLE^TM^ Select (Gibco).

All cell lines were grown in a humidified atmosphere at 37°C with 5% CO_2_ (Binder CO_2_ incubator C150).

### Construction of Azurin Recombinant Plasmid and Transfection Into Human MSC

Azurin coding sequence was obtained by gene synthesis following a codon optimization algorithm toward the human codon usage from the coding sequence from *P. aeruginosa* PAO1, to improve translation efficiency. Human codon optimized azurin (hazu) in fusion with the first 21 amino acids (aa) of the human tissue plasminogen activator (t-PA) (Qiu et al., 2000) was subcloned into a pVAX1-GFP vector by replacing the *GFP* gene, producing the recombinant pVAX-hazu plasmid. pVAX-GFP was constructed and produced as described elsewhere (Azzoni et al., 2007). The fidelity of the cloned sequence was evaluated by DNA sequencing. MSC were transfected with 10 μg of pVAX-hazu plasmid through microporation [Microporator MP100 (Neon/Invitrogen-Life Technologies)] according to Madeira et al. (2011); Sahin and Buitenhuis (2012). As a control, MSC were transfected with pVAX-GFP to assess the transfection efficiency. MSC conditioned media (CM) (MSC-CM) and cells were harvested at 72 and 96 h post-transfection. The expression and secretion of azurin were evaluated through Western blotting, and the percentage of GFP-positive cells was detected by flow cytometry (FACSCalibur, BD).

### Western Blotting

MSC-CM were collected at 96 h, mixed with loading buffer (Tris–HCl 62.5 mM, pH 6.8, 2.5% SDS, 10% glycerol, 0.002% bromophenol blue, and 5% β-mercaptoethanol), and boiled at 95°C for 5 min. Denatured samples were run on 15% polyacrylamide gel and transferred onto nitrocellulose membranes (*Trans-*Blot Turbo, BioRad). The membranes were incubated overnight with 1:2000 dilution of specific custom-made primary anti-azurin antibody (SicGen) (Bernardes et al., 2013), 1:2000 anti-GFP (Santa Cruz Biotechnology), or 1:1000 anti-GAPDH (Santa Cruz Biotechnology). Following incubation, the membranes were washed with PBS–tween-20 (0.5%) and probed with 1:2000 secondary antibody (Santa Cruz Biotechnology) during 1 h by shaking at room temperature. Proteins were detected through the addition of ECL reagent (Pierce) as a substrate and exposed and captured the chemiluminescence by Fusion Solo (Vilber Lourmat) equipment. For the cleavage of N-linked oligosaccharides, 10 μg of total protein in MSC-derived conditioned medium (MSC-CM) was mixed with 1 μl of Glycoprotein Denaturating Buffer (10×) and H_2_O, before boiling the sample for 10 min at 100°C. After briefly chilling on ice, 2 μl of GlycoBuffer (10×), 2 μl of 10% NP-40, and water were added to a final volume of 20 μl. Finally, 1 μl of PNGase F (New England Biolabs) was added and the mixture was incubated at 37°C for 1 h before analysis by Western blotting.

### Cancer Cell Proliferation Assay

Presto Blue^TM^ viability assay was used to determine proliferation of cancer cells upon treatment with MSC-CM. Cells were seeded on 96-well plates (Orange Scientific) at a density of 1 × 10^4^ and 2 × 10^4^ cells/well for MCF-7 and A549 cell lines, respectively. After 24 h, medium was exchanged with 100 μl of MSC-CM (keeping a baseline level of 50% cancer cells’ culture media: 0%, 10%, 25%, and 50% MSC-CM). Afterward, Presto Blue Reagent (ThermoFisher) was added to each well and incubated at 37°C for 2 h. Fluorescence was determined at the following wavelengths: 540 nm excitation and 590 nm emission. Untreated cells were used as control, in order to determine the relative cell proliferation of treated cells.

### Assessment of Cancer Cell Apoptosis

Cancer cell apoptosis was assessed using the Annexin V Apoptosis Detection kit (BD Sciences). Cells were plated on 6-well plates (Orange Scientific) at a density of 2 × 10^5^ and 1.5 × 10^5^ cells/well for MCF-7 and A549 cell lines, respectively. On the next day, medium was exchanged with MSC-CM (50% cancer cells’ culture media/50% MSC-CM). After 24 h incubation, cells were harvested and stained for Annexin V and propidium iodide (PI) by flow cytometry.

### Cancer Cell Invasion Assay

The ability of MSC to migrate toward tumor cells (tumor tropism) and cancer cell invasion was evaluated using CytoSelect^TM^ 24-Well Cell Migration with 8 μm pore size, coated with Matrigel. For tumor tropism experiments, 1.5 × 10^5^ lung (A549) and breast cancer (MCF-7) cells were cultured on 24-well plates and left overnight at 37°C and 5% CO_2_. MSC (4 × 10^4^) were incubated in the upper compartment of the culture chamber, placed on the wells, and left for 24 h at 37°C and 5% CO_2_. For cancer cell invasiveness experiments, 1.5 × 10^5^ A549 cells treated or untreated with MSC-CM were incubated in the upper compartment of the transwell, while culture medium (i.e., DMEM supplemented with 10% FBS) was added to the 24-well plates. Incubation was held at 37°C and 5% CO_2_ for 24 h. Non-migrated cells were removed from the upper side of the chamber’s filter with a cotton swab dipped in PBS and chambers were washed with PBS. Migrated cells were fixed in cold methanol (4°C) for 10 min. The membrane was removed with a scalpel and placed in a microscope glass, and cells were stained with DAPI and counted under a microscope (Zeiss). In each condition, 10 independent fields were counted, and the average of these fields was considered as the mean number of migrated cells per condition. Results are presented as the fold change in the number of cells migrated in comparison with the control condition where no cancer cells were added.

### Cancer Cell Migration Assay

A scratch assay was used to assess the migration of breast (MCF-7) and lung (A549) cancer cells *in vitro*, upon treatment with MSC-CM. Approximately 2 × 10^6^ cells were seed in 2-well culture inserts (Ibidi), to ensure reproducibility within conditions, on 24-well dish and cultured in growth medium for 72 h until approximately 70–80% confluence. Inserts were removed, and cells were treated with 250 μl of MSC-CM (keeping the proportion 50% MSC-CM/50% cancer cells’ culture medium). Cells were monitored over time by time-lapse recording and distance moved by the cells was determined by measuring the unoccupied scratch area (% of unoccupied area/h).

### Cytokine Quantification by ELISA

The levels of IL6, TGF-β, SDF1-α, and VEGF were measured in 100 μl of MSC-CM collected at 96 h with a sandwich ELISA kit, according to the manufacturer’s instructions (RayBiotech).

## Results

### Human MSC Are Able to Express and Secrete Azurin Without Cell Viability Impairment

Bone marrow (BM)- and umbilical cord matrix (UCM)-derived MSC cultured under xenogeneic (xeno)-free conditions were transfected by microporation with plasmid DNA (pVAX-hazu) encoding for a human codon optimized version of azurin coding sequence containing on its N-terminal a secretory sequence leading to the secretion upon protein synthesis (Qiu et al., 2000). Codon optimization was used considering that azurin is from a bacterial source and its efficiency of translation in animal cells, such as MSC, could be reduced. In parallel, MSC were transfected with a control vector, containing a green fluorescence protein (GFP) sequence (pVAX-GFP).

**FIGURE 1:**
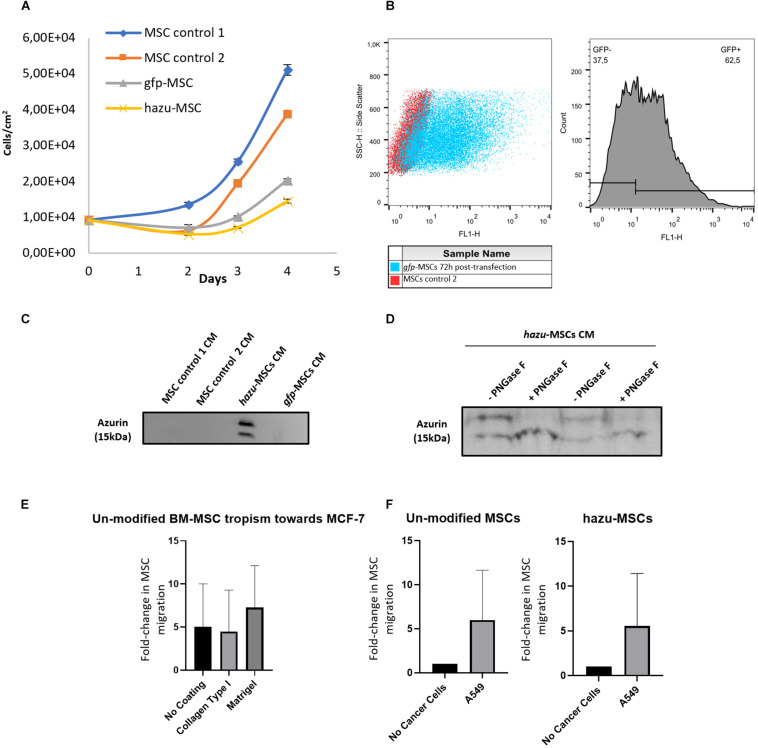
Engineering of mesenchymal stem/stromal cells (MSC) to express azurin. **(A)** MSC number per square centimeter after microporation. MSC non-microporated (control 1) (blue line), MSC microporation control were transfected without the plasmid DNA (control 2) (orange line), gfp-MSC were microporated with pVAX-GFP (gray line), and hazu-MSC were microporated with pVAX-hazu (yellow line). A total of 9.23 × 10^3^ cells per condition were initially microporated and counted at days 2, 3 and 4. Values are mean ± SD (*n* = 3). **(B)** Flow cytometry demonstrated that 50 to 60% of cell population was expressing GFP 72 h post-transfection. **(C)** Azurin is secreted by MSC to the conditioned media (CM) at 96 h after microporation. A representative image of Western blotting for one donor is depicted. **(D)** Ten micrograms of total protein from CM was incubated with PNGase F to remove *N*-linked oligosaccharides from glycoproteins. Western blotting image of MSC-CM from two independent donors is depicted. **(E)** Tumor tropism of un-modified bone marrow (BM)-derived MSC toward MCF-7 breast cancer cells. Results are presented as the fold change of migrated MSC toward tumor cells compared to negative control (migration toward culture media). **(F)** Comparison between tumor tropism rate of un-modified MSC and hazu-MSC toward A549. Results are presented as the fold change of migrated MSC toward tumor cells and the negative control (migration toward culture media).

Notably, azurin production has not induced alterations on MSC themselves, as we monitored cell viability over a 96-h period after cell microporation (Figure 1A). Non-transfected cells (control 1) displayed the highest cell number at day 2, 3, and 4, followed by cells microporated without DNA (control 2). Nevertheless, cells microporated with pVAX-GFP and pVAX-hazu entered the exponential growth phase with almost no differences between the groups. Flow cytometry demonstrated that 50 to 60% of the cell population was expressing GFP, 72 h post-transfection (Figure 1B), with a cellular recovery of 46% and a yield of transfection of 28% (comparable to 70, 40, and 30%, respectively, in Madeira et al., 2011). As negative controls, non-transfected cells (control 1) and microporated cells without DNA (control 2) were also evaluated.

After microporation, MSC were cultured for 96 h and the secreted azurin was detected in the CM by Western blotting (Figure 1C) (full membrane images are depicted in Supplementary Material 1). Specific bands around the expected MW of 15 kDa were observed only in the supernatants from MSC transfected with pVAX-hazu (hazu-MSC), which indicated that azurin was successfully expressed and released to the CM. However, it was possible to observe two bands corresponding to possible protein post-translational modifications. After treatment of MSC-derived CM (MSC-CM) with PNGase F (endoglycosidase that selectively removes N-glycans), only one band with more intensity was observed, which indicates that azurin is N-glycosylated in the CM of MSC-transfected cells (Figure 1D).

### MSC Preserve Tumor Tropism After Microporation

Human MSC have been described to be intrinsically tropic to tumor sites (Kidd et al., 2009), which is a central feature to their potential role as delivery vehicles for anticancer agents in cancer therapy. In this study, the *in vitro* tumor tropism properties of bone marrow-derived MSC (BM MSC) (three donors) toward a breast cancer cell line (MCF-7) were evaluated by a transwell migration assay using CytoSelect chambers with 8-μm pores. Aiming at a better mimicry of the *in vivo* microenvironment, we studied the effect of physiological barriers like collagen type I and Matrigel^TM^ as coatings on transwell chambers. Tumor cell lines were seeded on 24-well plates, and after 24 h, the upper chambers containing seeded MSC were added to each well at a MSC/tumor cells ratio = ¼;. In the control condition, no tumor cells were added (the corresponding medium volume was added instead). Tumor cells triggered invasion of BM MSC as compared to the negative control, and the specificity of this process seems to be improved by the presence of Matrigel (Figure 1E).

Cell microporation and transgene expression could potentially induce changes in the physiological properties of MSC. Therefore, we compared the tumor tropism rate of un-modified MSC and hazu-MSC toward A549 cells (Figure 1F). As shown, the expression of azurin does not impact the homing ability of these cells, and these results are supported by the characterization of CXCR4 (Supplementary Material 2), a known chemokine receptor associated with the tumor tropism properties of MSC (21.2% expression in control MSC versus 23.2% expression in hazu-MSC, assessed by flow cytometry).

### Cancer Cell Proliferation Decreases and Cell Death Increases Upon Treatment With hazu-MSC-CM

**FIGURE 2:**
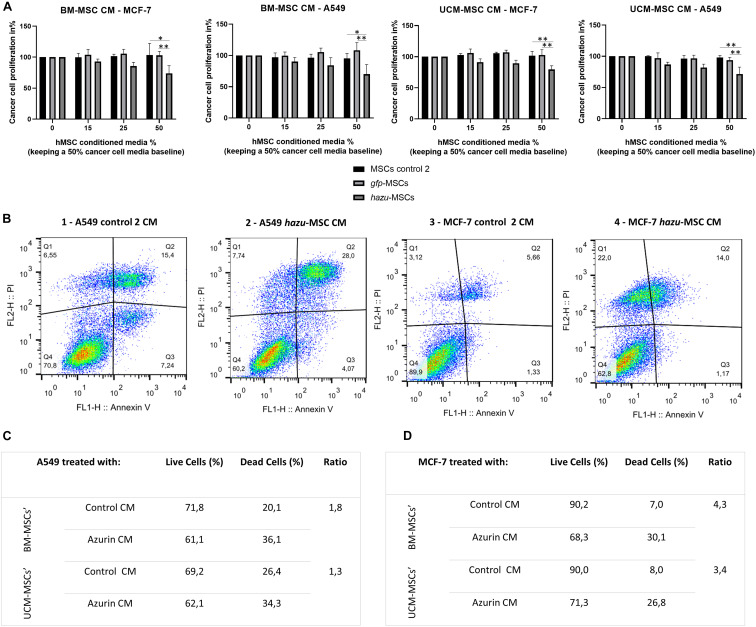
hazu-MSC CM inhibit cancer cell proliferation and induce cancer cell death *in vitro.* **(A)** Cytotoxicity and tumor cell viability were assessed by PrestoBlue in breast cancer (MCF-7) and lung cancer (A549) cell lines upon 24 h of exposition to conditioned media (CM) from MSC microporated with pVAX-hazu (hazu-MSC) (gray bars), pVAX-GFP (gfp-MSC) (light gray bars), or without DNA (microporation control 2) (black bars). MSC-CM was collected 96 h post-transfection. Due to differences in expansion media between cancer cells and MSC, MSC-CM concentration was variated (0–50%) while maintaining a level of cancer cells’ culture media at 50%. Untreated cells were exposed to media without CM, and their proliferation rate was admitted as 100% (*p*-values compare % of proliferation between gfp-MSC or hazu-MSC with MSC control 2; *n* = 4). **(B)** Annexin V expression detection after treatment with hazu-MSC’ CM, assessed by flow cytometry. Living cells are seen in the lower left quadrant, Annexin V (−)/ PI (−), [Q1]. The early apoptotic cells are shown in the lower right quadrant, Annexin V (+)/ PI (−), [Q2]. Advanced apoptotic or necrotic cells are seen in the upper right quadrant, Annexin V (+)/ PI (+), [Q3]. Annexin V (−)/ PI (+), [Q4] are cells in late necrosis or cellular debris. Panels 1 and 2 correspond to MCF-7 treated with control 2 MSC-CM and hazu-MSC-CM, respectively. Panels 3 and 4 correspond to A549 treated with control 2 MSC CM and hazu-MSC CM, respectively (*n* = 2). **(C)** Percentage of A549 live and dead cells based on flow cytometry results on annexin V expression, after treatment with MSC-CM and the ratio between dead cells treated with hazu-MSC’ CM or control MSC’ CM (*n* = 1). **(D)** Percentage of MCF-7 live and dead cells based on flow cytometry results on annexin V expression, after treatment with MSC-CM and the ratio between dead cells treated with hazu-MSC’ CM or control MSC’ CM (*n* = 1). Statistical differences are indicated with ^∗^*p* ≤ 0.05 and ^∗∗^*p* ≤ 0.01.

To investigate whether the secretome of azurin-producing MSC has an inhibitory effect on cancer cells’ growth and proliferation, tumor cell lines A549 and MCF-7 were subjected to increasing concentrations of CM from hazu-MSC cultures, harvested 96 h post-microporation. Since MSC and cancer cells were cultured in different culture media (MSC in StemPro^®^ MSC SFM XenoFree culture medium, whereas MCF-7 and A549 in DMEM high glucose supplemented with FBS), for this experiment, the concentration of MSC-CM was varied, while maintaining a baseline level of cancer cells’ culture medium at 50%. Cytotoxicity and tumor cell proliferation were assessed by using PrestoBlue after 24 h treatment with MSC-CM (Figure 2A). The results are presented in variation (%) of proliferation relatively to the control, where no MSC-CM was added (corresponding to 100% proliferation rate). The effect of hazu-MSC-CM seems to be inhibiting tumor cell proliferation, and this effect is more pronounced by increasing concentrations. On the other hand, CM retrieved from control MSC cause no change in the proliferation of both A549 and MCF-7 cell lines. The effect of hazu-MSC-CM induced an inhibition of 38.1% in A549 and an inhibition of 17.3% in MCF-7 with the highest concentration of CM (50% vol/vol). Moreover, we observed an average of 1.6- and 3.9-fold increase in the apoptotic levels of A549, assessed by flow cytometry (Figures 2B,C) and MCF-7 cells (Figures 2B,D), respectively, after treatment with hazu-MSC-CM when compared with the control CM.

### Cancer Cell Migration and Invasion Decrease Upon Treatment With hazu-MSC-CM

**FIGURE 3:**
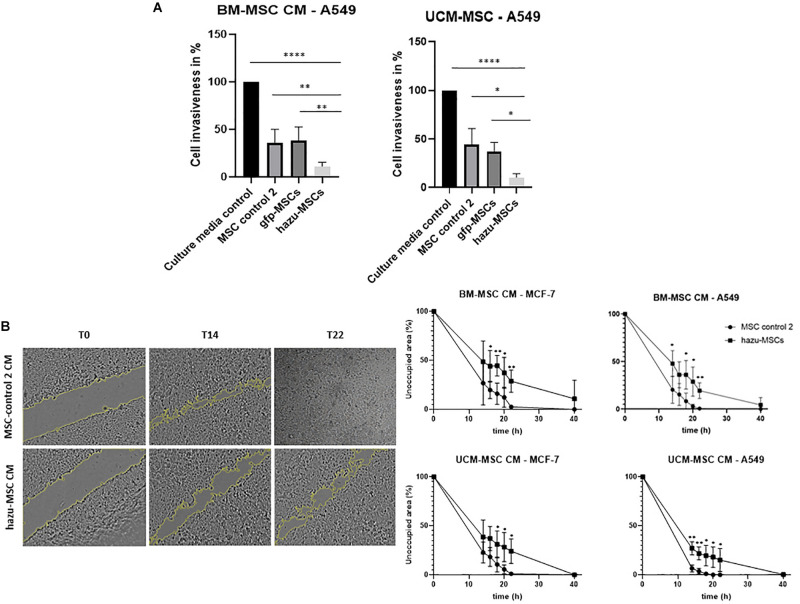
Inhibition of cancer cell invasion and migration by hazu-MSC’ CM *in vitro*. **(A)** A549 lung cancer cell invasion toward a chemoattractant (culture media supplemented with FBS) was evaluated in Matrigel invasion assays. Cells were treated with CM from gfp-MSC, hazu-MSC, MSC control 2, and cancer cell media (culture media control) during 24 h and migrated cells were quantified. Results are presented as the percentage of invasive cells compared to the control condition (*p*-values compare % of cancer cell invasiveness between hazu-MSC’ CM treatment and the remaining treatment conditions; *n* = 4). **(B)** Cell migration was estimated by means of a scratch assay and monitored by time-lapse microscopy. A549 and MCF-7 were treated with control 2 MSC’ CM or hazu-MSC’ CM, and the distances of migrated cells were measured at several time points: 0, 14, 16, 18, 20, 22 and 40 h (*p*-values compare % of unoccupied area between A549 and MCF-7 treated with hazu-MSC’ CM or MSC control 2 CM, at the same time point; *n* = 4). Statistical differences are indicated with ^∗^*p* ≤ 0.05, ^∗∗^*p* ≤ 0.01 and ^****^p ≤ 0.0001.

The antitumoral effects of hazu-MSC-CM are also extended to the impairment of cancer cell invasion. These experiments were performed with indirect co-cultures, in a transwell migration assay, by culturing cancer cells treated and un-treated with MSC-CM in invasion chambers coated with Matrigel. Results are given in the percentage of cancer cell invasion in comparison to the control condition where cancer cells were treated with culture medium only (i.e., without MSC-CM). By analyzing the results, we can hypothesize that the naïve MSC’ secretome by itself has an impact in reducing cancer cell invasion, and this effect is enhanced by the presence of azurin to a notorious extent (close to 20% invasive cells compared to control) (Figure 3A).

Cell migration in cancer cells is also affected by treatment with hazu-MSC-CM. Cell migration was estimated by means of a scratch assay and monitored by time-lapse microscopy. The distances of migrated cells were measured over several time points and the results show that treatment with CM from hazu-MSC induced a delay on cancer cell migration and repairment of the scratch area (Figure 3B). Twenty hours after treatment, the percentage of unoccupied area for A549 treated with hazu-MSC-CM was 23.4%, compared to 1.4% unoccupied area for A549 treated with CM from control 2 MSC. Regarding MCF-7, 32.8% was observed for cells treated with hazu-MSC-CM and 8.9% was observed for cells treated with CM from control 2 MSC.

### Secretion of Cytokines Involved in Tumor Progression by Engineered MSC Quantified by ELISA

**FIGURE 4:**
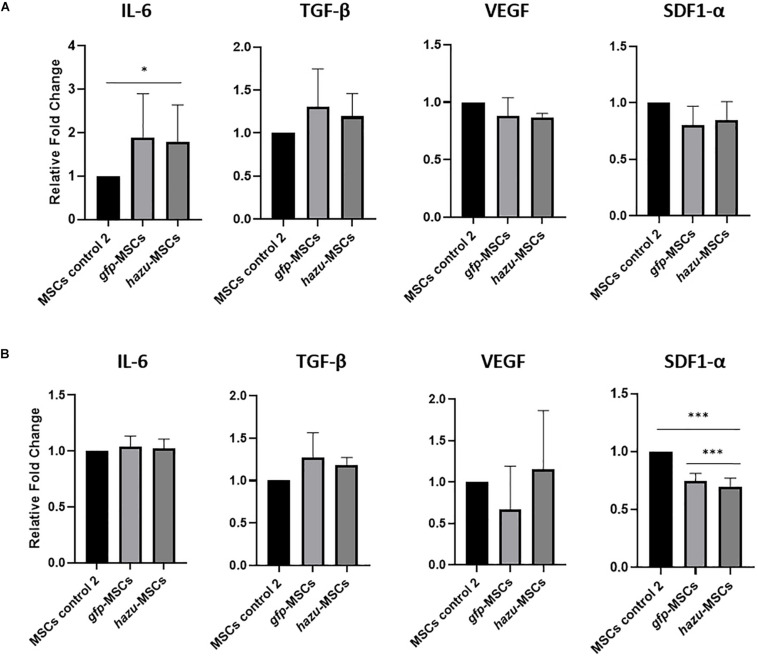
Evaluation of MSC expression profile on cytokines relevant in cancer progression. The levels of inteuleukin-6 (IL6), vascular endothelial growth factor (VEGF), stromal derived factor 1 alpha (SDF1-α), and transforming growth factor beta (TGF-β) were analyzed by ELISA in MSC-CM before and after microporation with pVAX-hazu and pVAX-GFP. The results are given in the relative fold change of cytokine expression relatively to MSC-CM in the control condition (control 2) (*p*-values compare fold change between gfp-MSC and hazu-MSC conditions with MSC control 2; *n* = 3). **(A)** Cytokine expression profile in BM MSC-CM. **(B)** Cytokine expression profile in UCM MSC-CM. Statistical differences are indicated with ^∗^*p* ≤ 0.05 and ^∗∗∗^*p* ≤ 0.001.

To get insights into the antitumoral effects induced by hazu-MSC-CM, namely, if these are due to a crosstalk between the induced azurin expression and the native secretome of MSC, we evaluated the expression of four cytokines expressed by MSC that have been described to have a role in MSC interaction with cancer cells: inteuleukin-6 (IL6) (Kidd et al., 2009), vascular endothelial growth factor (VEGF) (Vera et al., 2019), stromal derived factor 1 alpha (SDF1-α) (Liu et al., 2010), and transforming growth factor beta (TGF-β) (Markell et al., 2010). To this end, we analyzed the concentration of these factors in MSC-CM by ELISA before and after microporation with pVAX-hazu and pVAX-GFP (Figure 4). The results are given in the relative fold change of cytokine expression relatively to MSC-CM in the control condition (control 2, i.e., MSC microporated without DNA). The microporation process seems to be inducing a general response in the expression of such cytokines, by decreasing their relative concentration. However, no significant differences were observed between the hazu-MSC-CM and gfp-MSC-CM, which might suggest that the effects observed in cancer regression can be due to the engineered expression of azurin independently.

## Discussion

One of the major challenges of developing more effective cancer therapies concerns the specific delivery of anticancer drugs to the tumor site. In this context, human MSC have been recently considered for cell-based therapies for cancer, due to their ability to migrate specifically and to incorporate within tumors, their low immunogenicity and the fact that these cells are relatively easy to isolate, culture, and manipulate (Xie et al., 2013; Hofer and Tuan, 2016; Gao et al., 2016; Brewster et al., 2018; Mathew et al., 2019; Rifai et al., 2019). Altogether, these features turn MSC into exciting therapeutic candidates as drug delivery tools toward cancer. In the perspective of cell-based therapies, MSC do not only potentially solve the drug delivery specificity problem but also allow for the heightening of the drug compound’s half-life in the organism, as well as a lower dosage and less repeated injections to potentially achieve meaningful responses (Elman et al., 2014).

Furthermore, MSC demonstrate a strong paracrine effect resulting from the high levels of bioactive molecules they secrete in response to their microenvironment. The panoply of factors produced by these cells is highly context dependent, being able to be modulated *in vitro*. For this reason, MSC’s secretome, either in the format of CM or as purified extracellular vesicles (EVs), has been explored as a cell-free approach in several applications in regenerative medicine (Keshtkar et al., 2018; Eleuteri and Fierabracci, 2019). Despite the potential benefits of using MSC as a cell delivery system, studies have reported the supportive role of MSC in the progression of tumor density and metastasis, while others have shown antitumor effects both *in vitro* and in different models of cancer (Devarasetty et al., 2017; Liang et al., 2020; Xia et al., 2020). The conflicting data in the literature may hamper the establishment of cell therapies for cancer based on non-modified MSC since the therapeutic safety of such approach might be jeopardized (Rahmatizadeh et al., 2019).

The availability of genetic engineering tools may potentiate MSC as living factories of antitumoral proteins for cancer therapy. In this study, we genetically engineered human MSC, through non-viral methods, toward the production and secretion of the antitumoral protein azurin. Azurin, originally produced by *P. aeruginosa*, has a complex anticancer mechanism of action, targeting several independent pathways critical for tumor progression. These features allow a much broader action of azurin regarding the tumor types that it can target, while also supporting the prevention of tumor resistance (Bernardes et al., 2014, 2018, 2016). We engineered a recombinant plasmid containing the azurin coding sequence and an engineered secretory sequence that provides a signal for translocation of recombinant proteins into the lumen of the endoplasmic reticulum (ER), for transport through the ER and Golgi apparatus to the extracellular environment (Qiu et al., 2000). To the best of our knowledge, this study is the first to combine a stem cell-based approach to deliver a protein originated in bacteria for anticancer therapies.

In what concerns clinical trials studying the use of genetically engineered MSC as cell therapy for cancer, three first-in-human studies are being conducted. The phase I/II TREAT-ME trial (NCT02008539) assesses the safety and efficacy of autologous MSC genetically modified with a retroviral vector expressing tyrosine kinase and subsequent ganciclovir infusions in patients with gastrointestinal adenocarcinoma. The results known so far demonstrated safety and tolerability in treated patients, with preliminary signs of efficacy in terms of clinical stabilization of disease (von Einem et al., 2019). The TACTICAL trial (NCT03298763) assesses the safety and efficacy of allogeneic umbilical cord-derived MSC transduced with lentivirus to express TRAIL as a first-line therapy in conjunction with chemotherapy in patients with metastatic adenocarcinoma of the lung. Finally, a study employing MSC genetically modified with a plasmid vector to produce IFN-β (NCT02530047) assesses the safety and efficacy in patients with advanced ovarian cancer. Many other studies have recently reported engineered versions of MSC aimed to treat cancer at the preclinical level.

Over the last years, significant efforts have been made to address the limitations of MSC in early clinical trials, namely, by using genetic engineering tools to improve the therapeutic potential of these cells (Nowakowski et al., 2016). Despite the advantages of employing non-viral gene delivery methods, to date, the majority of conducted clinical trials based on genetically engineered MSC are relying on the use of viral methods. Although transduction efficiency is higher, issues regarding vectors safety and manufacturing have encouraged the implementation and optimization of non-viral based techniques such as microporation. The method used in this study is based on previous studies from our group (Madeira et al., 2011), aiming at a cell transfection with high efficiency without compromising cell viability and recovery. Regarding the percentage of GFP-positive cells, herein we obtained 60%, a cellular recovery of 46% and yield of transfection of 28% (70, 40, and 30%, respectively, in Madeira et al., 2011). hazu-MSC supernatants were collected at 96 h and azurin was detected by Western blotting. Besides the expected azurin, it was possible to observe a second band corresponding to a post-translationally modified protein, that was later identified as a glycosylated azurin after treatment with PNGase F. This brings us to hypothesize that the activity of this glycosylated form of azurin may differ from the native protein. Therefore, in future studies, it would be important to characterize this modified protein in terms of structure, functionality, and antitumoral activity. Although it is expected that MSC continue to secrete azurin for longer than 96 h, we anticipate that after culturing cells for such a long time period, their CM will be exhausted from key nutrients and MSC will likely secrete proteins and factors responding to metabolic stress, which makes the interpretation of the results difficult herein.

We tested the effect of hazu-MSC secretome in tumor progression by exposing MCF-7 and A549 cells to increasing concentrations of engineered MSC-derived CM. The plenitude of hazu-MSC produced factors inhibited 17.3 and 38.1% tumor proliferation in MCF-7 and A549, respectively, with the highest concentration of CM tested (50%, vol/vol) compared to MSC microporated with pVAX-GFP and MSC microporated with no DNA (control 2), where no inhibition was observed. In this experiment, we varied the concentration of MSC-CM, while maintaining a baseline level of cancer cells’ culture medium at 50%. Thus, the effects observed in cancer proliferation are not associated with the medium change or the lack of FBS components. Along with a decrease in cancer cell proliferation, an increase in cancer cell apoptosis was observed. These results are in agreement with the anticancer properties of azurin, as previously mentioned (Yamada et al., 2004). Moreover, upon treatment with hazu-MSC-CM, a decrease in invasion through Matrigel for the A459 invasive cell line (Wang et al., 2017) and a decrease in cell migration were observed for both cancer cell lines. In previous work from our group, we demonstrated that bacterial produced azurin is able to interfere with pro-tumorigenic and proliferative signaling pathways FAK, Src, and AKT, by attenuating the phosphorylation levels of these proteins in lung (Bernardes et al., 2016) and breast cancer cell lines (Bernardes et al., 2013). After treatment with azurin, besides a decrease of FAK and Src phosphorylation, we observed a 44–66% reduction of cancer cell invasion through Matrigel (Bernardes et al., 2013). In what concerns lung cancer cells, azurin was also associated with attenuated phosphorylation levels of Src Y416, Akt S473, and PI3K, which correlated to a 30% reduction in the invasive capacity of the cancer cells by around 30% (Bernardes et al., 2016). In this context, further studies should focus on the interaction between MSC-produced azurin and the activation of such signaling pathways.

MSC are emerging as promising anticancer agents, fundamentally due to their innate tropism toward proinflammatory environments, such as the tumor microenvironment in both primary and metastatic sites (Oieni et al., 2019). In this context, we demonstrated the migratory capacity of hazu-MSC toward MCF-7 and A549 cancer cell lines through indirect co-cultures. The results demonstrated no differences in the migratory potential of engineered when compared to unmodified cells. Furthermore, we evaluated the expression of four cytokines expressed by naïve MSC that play a pivotal role in the hallmarks of cancer progression in processes such as cancer cell proliferation, invasion, migration, angiogenesis, apoptosis, and development of metastases (Maskarinec et al., 2020). Microporation seems to be inducing an effect in the expression of such cytokines to a certain extent; however, we observed no significant differences between engineered MSC and naïve MSC, which may suggest that the results observed in cancer regression might be associated to the expression of azurin independently, rather than due to a crosstalk of azurin and the naïve MSC secretome.

The majority of the studies evaluating the effect of naïve MSC on tumor development employ MSC from the BM, the UCM, and the AT (Rahmatizadeh et al., 2019; Liang et al., 2020; Xia et al., 2020). When analyzing the outcome from these studies, it seems to be a conspicuous pattern of tumorigenicity, with BM MSC being more pro-tumorigenic and UCM MSC being more tumor suppressive. This pattern seems to be more pronounced when evaluating breast cancer, the most popular type of cancer tested with MSC cytotherapy (Christodoulou et al., 2018). For this reason, in the present study, all experiments were validated using MSC isolated from two different donors of two tissue sources, BM and UCM.

Although BM has been the main source for MSC isolation, the harvest of BM is a highly invasive procedure and the number, differentiation potential, and maximal life span of BM MSC decline with increasing age (Kern et al., 2006). In this regard, a significant advantage of the neonatal tissues, such as the UCM, as sources of MSC is that they are readily available, thus avoiding invasive procedures and ethical problems associated with adult tissues, and several studies have reported superior proliferative capacity, life span, and differentiation potential over BM MSC (Kern et al., 2006). Considering the ease of harvest, culture, and transfection of MSC, the use of autologous cells may be realistic. However, the number and quality of MSC differ from patient to patient, making the quantification of the therapeutic effect difficult to interpret. Therefore, the use of allogenic MSC from healthy donors would allow greater cell numbers of better characterized cells (Loebinger et al., 2009). Moreover, envisioning an MSC cell line that stably expresses the transgene could overcome some issues related to the translation of MSC cytotherapy to a clinical setting. Therefore, as ongoing work of our group, the establishment of a stable hazu-MSC cell line represents a more flexible system in terms of both manufacturing and therapeutic perspectives (cell-based product or cell-free approach based on CM).

## Conclusion

In this study, we were able to engineer MSC from two different tissue sources (BM and UCM) to express and secrete a human codon optimized version of an antitumoral bacterial protein, secreting it into the extracellular environment. When testing the CM retrieved from hazu-MSC, we observed a decrease in cell proliferation, migration, and invasion of breast (MCF-7) and lung (A549) cancer cell lines. In addition, an increase in cell death was observed for both cell lines.

All in all, the results presented here add to the arsenal of cell-based therapies for cancer, using the natural tumor-targeting properties of MSC and the broad anticancer functional activity of azurin.

## Data Availability Statement

All datasets generated for this study are included in the article/Supplementary Material.

## Author Contributions

NB and CS conceived and designed the study. MS and NB performed the experiments. MS, NB, and CS wrote the manuscript. All authors critically read and approved the final manuscript.

## Conflict of Interest

The authors declare that the research was conducted in the absence of any commercial or financial relationships that could be construed as a potential conflict of interest.
