# Supplementary figures and images for: Conditioned Medium From Azurin-Expressing Human Mesenchymal Stromal Cells Demonstrates Antitumor Activity Against Breast and Lung Cancer Cell Lines
Source: Front Cell Dev Biol. 2020 Jul 9;8:471. doi: 10.3389/fcell.2020.00471 (PMC7363770; doi:10.3389/fcell.2020.00471)

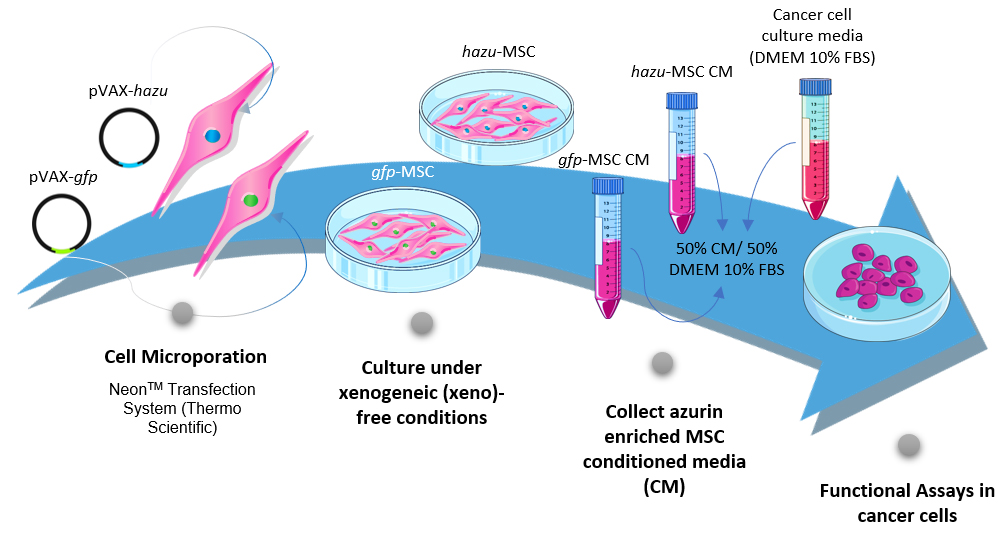

Supplement: Supplementary file 1 [file Image_1.JPEG]

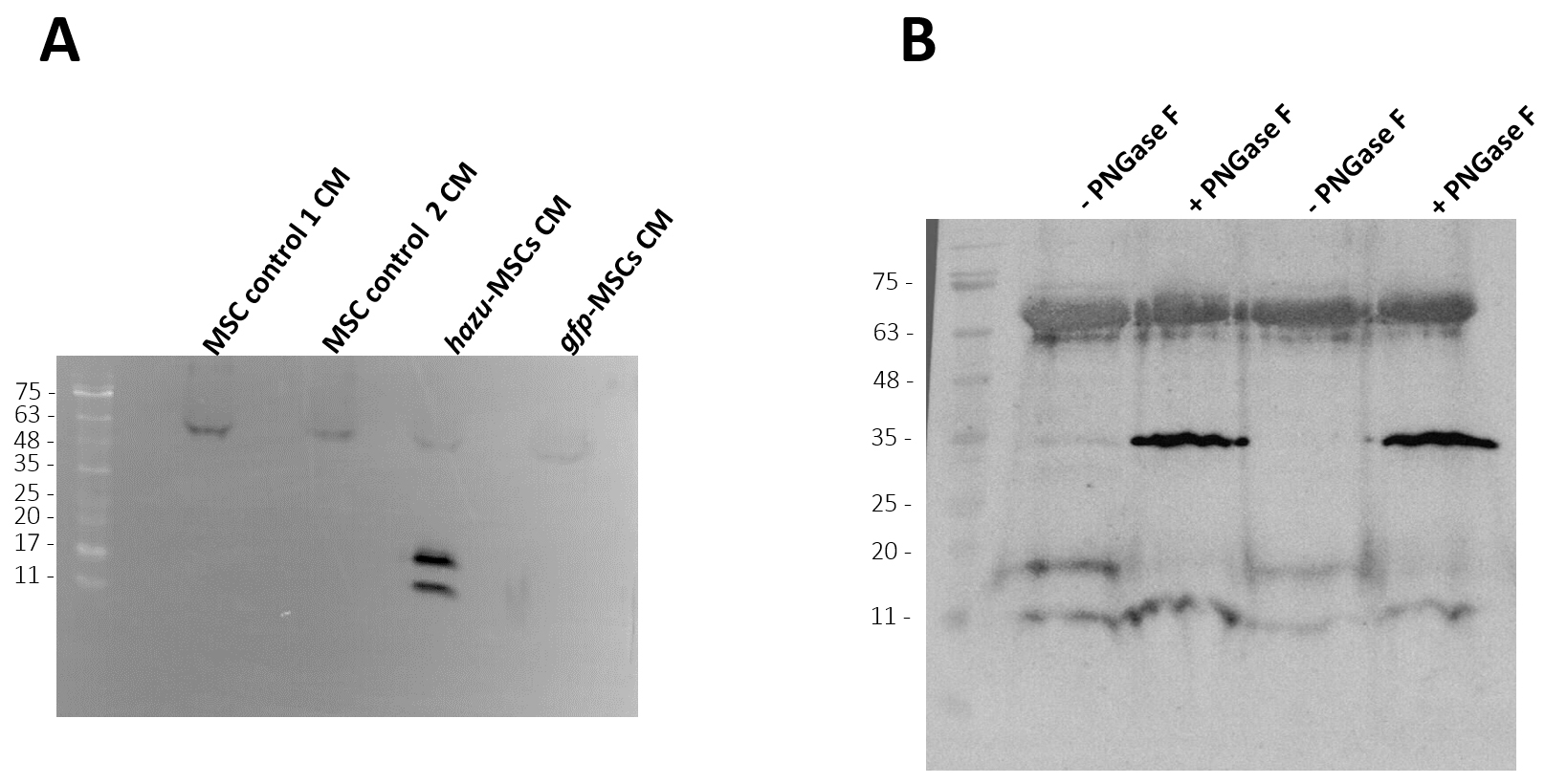

Supplement: Supplementary file 2 [file Image_2.JPEG]

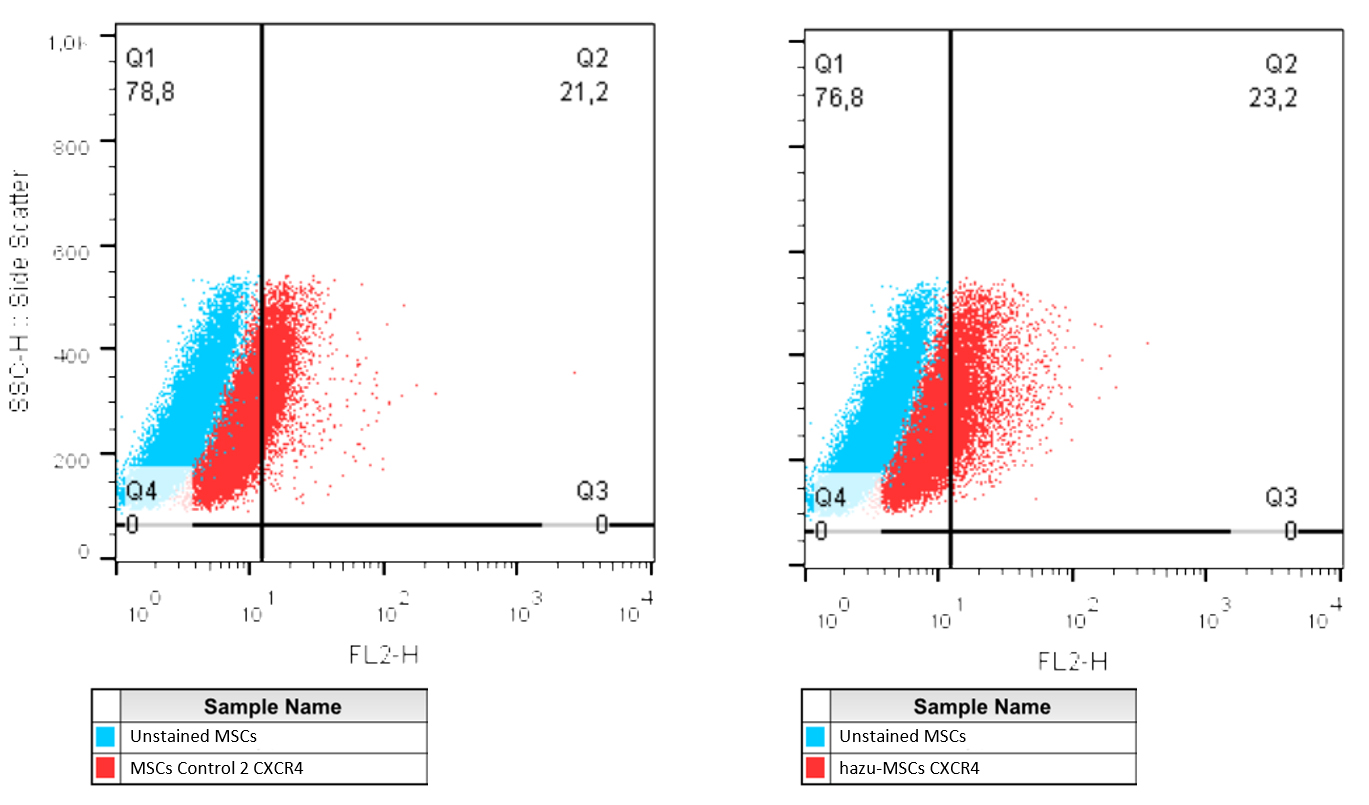

Supplement: Supplementary file 3 [file Image_3.JPEG]
